# Neuroprotective Effect and Possible Mechanisms of Ginsenoside-Rd for Cerebral Ischemia/Reperfusion Damage in Experimental Animal: A Meta-Analysis and Systematic Review

**DOI:** 10.1155/2022/7650438

**Published:** 2022-09-01

**Authors:** Ai-fang Zhou, Ke Zhu, Pei-min Pu, Zhuo-yao Li, Ya-yun Zhang, Bing Shu, Xue-jun Cui, Min Yao, Yong-jun Wang

**Affiliations:** ^1^Spine Disease Institute, Longhua Hospital, Shanghai University of Traditional Chinese Medicine, Shanghai 200032, China; ^2^Rehabilitation Medical College, Shanghai University of Traditional Chinese Medicine, Shanghai 201203, China; ^3^Department of Traditional Medicine, Longhua Hospital, Shanghai University of Traditional Chinese Medicine, Shanghai 200032, China; ^4^Department of Orthopedics and Traumatology, Longhua Hospital, Shanghai University of Traditional Chinese Medicine, Shanghai 200032, China; ^5^Shanghai University of Traditional Chinese Medicine, Shanghai 201203, China

## Abstract

Ischemic stroke, the most common type of stroke, can lead to a long-term disability with the limitation of effective therapeutic approaches. Ginsenoside-Rd (G-Rd) has been found as a neuroprotective agent. In order to investigate and discuss the neuroprotective function and underlying mechanism of G-Rd in experimental animal models following cerebral ischemic/reperfusion (I/R) injury, PubMed, Embase, SinoMed, and China National Knowledge Infrastructure were searched from their inception dates to May 2022, with no language restriction. Studies that G-Rd was used to treat cerebral I/R damage *in vivo* were selected. A total of 18 articles were included in this paper, and it was showed that after cerebral I/R damage, G-Rd administration could significantly attenuate infarct volume (19 studies, SMD = −1.75 [−2.21 to − 1.30], *P* < 0.00001). Subgroup analysis concluded that G-Rd at the moderate doses of >10- <50 mg/kg reduced the infarct volume to the greatest extent, and increasing the dose beyond 50 mg/kg did not produce better results. The neuroprotective effect of G-Rd was not affected by other factors, such as the animal species, the order of administration, and the ischemia time. In comparison with the control group, G-Rd administration could improve neurological recovery (lower score means better recovery: 14 studies, SMD = −1.50 [−2.00 to − 1.00], *P* < 0.00001; higher score means better recovery: 8 studies, SMD = 1.57 [0.93 to 2.21], *P* < 0.00001). In addition, this review suggested that G-Rd *in vivo* can antagonize the reduced oxidative stress, regulate Ca^2+^, and inhibit inflammatory, resistance to apoptosis, and antipyroptosis on cerebral I/R damage. Collectively, G-Rd is a promising natural neuroprotective agent on cerebral I/R injury with unique advantages and a clear mechanism of action. More clinical randomized, blind-controlled trials are also needed to confirm the neuroprotective effect of G-Rd on cerebral I/R injury.

## 1. Introduction

Ischemic stroke is the leading cause of hospitalization for cerebrovascular disease, accounting for 85% of all stroke incidences [1]. It is associated with a variety of complications, including insomnia, depression, or poststroke dementia, which can lead to adverse outcomes [2–4]. As a leading cause of high morbidity, mortality, and disability rate, stroke imposes a severe financial and psychological burden on patients and families worldwide [5]. Making an urgent therapy is the most important and effective hotspot. Yet now, to achieve neuroprotection, majority of the therapeutic approaches for acute ischemic stroke are to recanalized the occluded arteries [5]. Currently, thrombolysis with recombined tissue plasminogen activator and thrombectomy are effective treatments [6, 7]. However, limited by a narrow therapeutic window and the risk of hemorrhagic complications, these methods only can be used in the minority [8, 9]. Just as importantly, cerebral reperfusion caused secondary damage to the brain can also lead to severe adverse reaction [10].

When acute ischemic stroke happened, blood flow sharply decreased which gives rise to a rapid increasing in the production of reactive oxygen species (ROS) [11, 12]. Early restoration of blood supply can salvage ischemic and hypoxic tissue, but reperfusion itself also can cause cerebral ischemia/reperfusion (I/R) damage [13, 14]. Most importantly, ROS are produced and bursts after reperfusion [15]. Excess ROS is the main cause of oxidative stress and one of the major hazards leading to the direct neuron damage [11, 16–18]. Oxidative stress triggered nuclear factor kappa-light-chain-enhancer of the activated B cell (NF-*κ*b) signaling pathway, leading to nucleotide-binding oligomerization domain-like receptor 3 (NLRP3) activation, those which contribute to the blood-brain barrier (BBB) damage, inflammatory environment formation, neuron apoptosis, and pyroptosis [17, 19–22]. Meanwhile, the occurrence of oxidative stress further attacks carbohydrates, lipids, proteins, nucleic acid, and release of Ca^2+^ from intracellular stores [23]. In addition, this microenvironment in turn further exacerbates oxidative stress [24, 25]. Thus, establishing a balance of ROS generation and consumption to attenuate I/R injury may be an effective therapeutic strategy.

**Figure 1:**
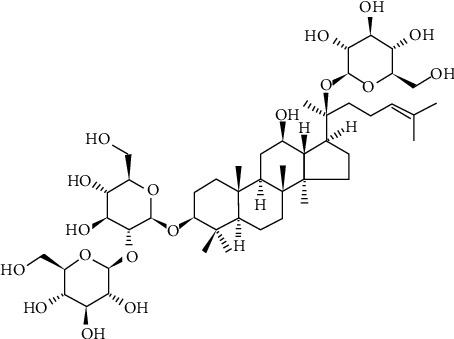
Chemical structures of G-Rd. G-Rd: ginsenoside-Rd.

After thousands of years of practice, traditional herbal extracts and its effective components are used worldwide as drug to prevent and treat ischemic stroke which has been collected both *in vivo/vitro* and in clinical application. Ginsenoside-Rd (G-Rd) (the chemical structure is shown in Figure 1), as a main bioactive saponin, belongs to the protopanaxadiol group [26]. G-Rd is an important metabolite in the transformation pathway of protopanaxadiol-type ginsenosides in human intestine [27]. To date, G-Rd can be obtained from structurally similar ginsenoside-Rb1 and ginsenoside-Rc by microbial-based biotransformation and enzymatic transformation [28, 29]. G-Rd has outstanding advantages in multisite and multitarget global regulations. Extensive studies showed G-Rd with multiplied pharmacological properties possesses a broad spectrum of therapeutic effects on the central nervous system [30, 31]. G-Rd directly makes contribution to the nuclear factor erythroid-2-related factor 2 (Nrf2) antioxidant pathway to promote the ability of eliminating ROS to inhibit lipid peroxidation [22, 32, 33]. Besides, G-Rd plays an anti-inflammatory role in Alzheimer's disease [34]. Furthermore, G-Rd with highly lipophilic ability can spread through biological membranes and BBB easily [31, 35]. Currently, preclinical studies have confirmed the effectiveness of G-Rd in the treatment of cerebral I/R, and then, G-Rd treatment of cerebral I/R has entered the second phase of clinical trials [31, 36, 37]. Its neuroprotective function has attracted an increasing attention. In order to review the beneficial effects of G-Rd on I/R damaged animal models and summarize the underlying molecular mechanisms, a comprehensive systematic review was performed.

## 2. Methods and Materials

The Preferred Reporting Items for Systematic Reviews and Meta-Analyses (the PRISMA) statement was selected to report for this systematic evaluation and meta-analysis [38].

### 2.1. Search Strategy

The following electronic databases were retrieved from their inception to May 2022 to identify relevant *in vivo* studies without language limitations: PubMed, Embase, China National Knowledge Infrastructure, and SinoMed. The lists of references included in this study are screened to identify if there are any other relevant studies. MeSH terms such as “brain anoxia ischemia”, “brain hypoxia ischemia”, “brain ischaemia”, “brain ischaemics”, “brain ischemia”, “brain ischemias”, “cerebral anoxia ischemia”, “cerebral ischaemia”, “cerebral ischaemia hypoxia”, “cerebral ischemia”, “cerebral ischemias”, “ischemic stroke”, “ischemized reperfusate”, “ischemized reperfusion injury”, “reperfusion injury”, “stroke”, “ginsenoside”, “ginsenosides”, and “ginsenoside Rd” were used. These search terms were translated into Chinese to be searched in Chinese databases. Supplementary material contains the PubMed database search strategy.

### 2.2. Inclusion Criteria

All controlled studies evaluating the neuroprotection effect and discussing the possible mechanisms of G-Rd on brain I/R damage in animal models were included. Specify the following inclusion criteria in advance to prevent bias (1) based on an animal experiment, no restriction on animal species, gender, age, weight, and sample size; (2) involve a focal cerebral I/R damage model, caused by transient or permanent middle cerebral artery occlusion (MCAO); (3) the experimental group was treated with G-Rd monotherapy in no restriction on dosage, mode, and time of initial treatment; (4) the control group was administered by saline, vehicle, or positive control drug or no treatment; (5) have one of the following outcomes available: infarct volume, neurological function score (NFS), and biochemical examinations.

### 2.3. Exclusion Criteria

The following exclusion criteria were also prespecified: (1) reviews, comments, case reports, editorials, clinical articles, and *in vitro* studies; (2) nonfocal brain I/R model, adopting global models (e.g., bilateral common carotid occlusion), traumatic models, or only hypoxic ischemic models; (3) the experimental group was intervened of G-Rd combined with some other drugs; (4) absence of control group; (5) outcome measures are not included in the literature; and (6) duplicated publications.

### 2.4. Selection and Data Extraction

Two reviewers (ZAF and ZK) screened the abstracts and full texts of the included literature and excerpted the following details independently: (1) the first author's name, the publication year, methods of establishing ischemia model, and ischemia duration; (2) the characteristics of animals, such as the number, the species, the gender, and the weight of animals; (3) treatment information including dosage, timing, and the route of G-Rd delivery; and (4) outcome assessment. Disregarding the outcomes presented at different time points of each animal in the experiment, only extracted the data at the last time point. If the research results were incomplete or only showed in the form of graphs, the authors were e-mailed for these data, and if a response was not received, the data was got from the graphs using Engauge Digitizer 11.1 commercial software. If several independent experiments are carried out in a paper, it was broken down into several complete sections. These two reviewers extracted relevant data from the papers independently to avoid errors. Take the average data of two when the error was within the acceptable range (error ≤ 1%average data). If not, the third one (YM) shall reextract the data and then use the average of two data which were more closely related.

### 2.5. Quality Assessment

Study quality was evaluated by the Collaborative Approach to Meta-Analysis and Review of Animal Data in Experimental Studies (CAMARADES), a ten-item modified scale [39]. Two investigators (ZYY and LZY) independently evaluated the methodological quality of the included literature according to the following list: (1) published in a peer-reviewed journal; (2) temperature control statement; (3) randomization to treat or control group; (4) allocation concealment; (5) outcome assessed blinding; (6) no obvious intrinsic neuroprotective effect of anesthetic; (7) appropriate animal model such as aged, diabetic, or hypertensive; (8) sample size estimation; (9) compliance with animal welfare regulations; and (10) declared any potential conflict of interest. A ten-item (1 point for each item) was included in the modified scale, and an aggregate quality score was obtained for each study. If the weighted kappa (*K*_w_) value was >0.75, the quality assessment was accepted, consulting with the corresponding author to solve any disagreements.

### 2.6. Statistical Analysis

Different scales were used in a different study to assess the same outcome index, so the standardized mean difference (SMD) with 95% CI was used in this analysis. The *I*^2^ test was used to assess the statistical heterogeneity of the included studies. *I*^2^ > 50% means significant heterogeneity exists, and then, the random-effect model test was conducted. Or then, the fixed-effect model test was selected. The factors modifying on the infarct volume were explored through the source of heterogeneity of subgroup analysis. *P* ≤ 0.05 was considered statistically significant. Review Manager version 5.4 software was used for data analyses.

### 2.7. Publishing Bias

Publishing bias means that the published research literature does not systematically or comprehensively represent the completed research in the field. Therefore, in order to further test for the publishing bias, funnel plot and Egger's test were used in this meta-analysis stage.

## 3. Results

### 3.1. Selection of Studies

**Figure 2:**
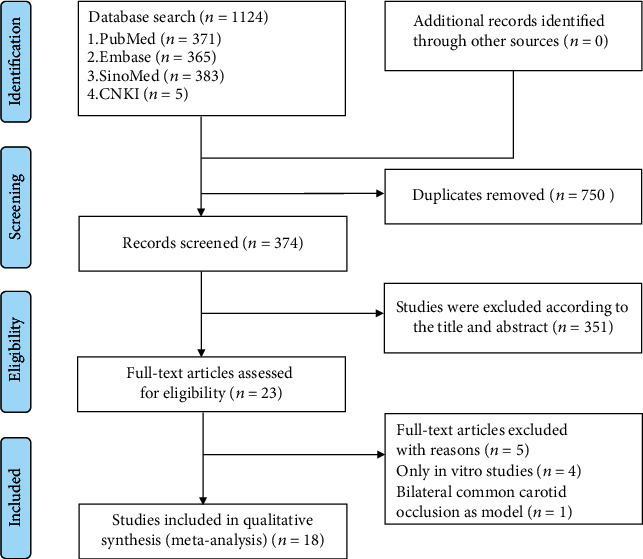
Summary of the literature identification and selection process.

The process of screening is summarized in Figure 2. In total, 374 unique references were identified by searching electronic databases and removing the duplicates. 351 papers were excluded after going through the titles and abstracts. For the reason: the studies of solely *in vitro*, 4 articles were deleted from the remaining articles by reading the full text [40–43]. Wan et al. used a model of bilateral common carotid occlusion which was a nonfocal brain I/R model so that it was excluded [44]. Eventually, 18 articles were obtained and assessed these for eligibility [22, 33, 35, 45–59].

### 3.2. The Characteristics of the Included Studies

All of these studies were conducted in China and reported in English except for three studies which were published in Chinese. The animal species included Wistar rats [48], C57BL/6 mice [22, 58], and Sprague-Dawley rats [33, 35, 45–47, 49–53, 57, 59]. In order to keep the baseline status of experimental animals consistent and to be studied independently, Ye et al.'s study was split into five complete parts: dose-response study, therapeutic window study, permanent ischemia study, older study, and female study [45]. Ye et al. were separated into three parts, including dose-response study, therapeutic window study, and sustained neuroprotection study [58], while in Ye et al.'s article, the authors introduced the protection conferred by G-Rd in two parts, including its sustained effects [35]. All the animals included were male except in the Ye et al.'s female study. Most of the studies were transient MCAO models, with cerebral artery occlusion varied from 1 to 2 hours. While Ye et al. reported a study in permanent MCAO. All studied animals conditioned with G-Rd by intraperitoneal injection. The dosing of G-Rd treatment varied substantially, five of the included studies performed a dose gradient study of G-Rd [33, 45, 47, 55, 58]. And single-dose administration was conducted in the remaining. Moreover, the duration of G-Rd intervention ranged from 3 days before ischemia to 1 day after the ischemia stroke happened [48, 55]. For comparison, in some studies, G-Rd also was tested and compared/combined with edaravone and PBN, LY294002, and MG132 [45, 50, 55] (Table 1).

### 3.3. Risk of Bias within Studies

The quality score of the included studies ranged from 3 to 7 out of 10 points (Table 2). Consensus was built on 100% with *K*_w_ = 0.89. Of whom, six studies received more than 5 points. All the included studies were published in peer review, 16 studies illustrate the control of temperature. Randomization and blinded assessment were reported in 11 and 8 studies, respectively, but none of them described the sample-size calculation and allocation concealment. 13 studies reported without significant neuroprotective activity from anesthetics, while others did not describe which anesthetic agent they were used. 8 studies declared without potential conflicts of interest. Moreover, only 3 studies stated they were in compliance with animal welfare laws. In terms of compliance with appropriate model animals, 2 studies pointed out they researched on older or/and female animals. As proposed by Bederson et al. [60], 5 studies pointed out a completed stroke model was identified with forelimb flexion, whereas Du's study described with score of 2-3 according to Longa scale is considered as a successful stroke model.

### 3.4. The Effective of G-Rd for Cerebral Infarct Volume in Cerebral I/R Injury and Meta-Analysis

**Figure 3:**
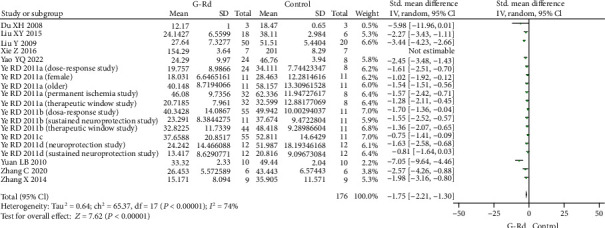
The pooled estimate of G-Rd on decrementing cerebral infarct volume after cerebral I/R damage. G-Rd: ginsenoside-Rd; I/R: ischemia/reperfusion.

There were 12 studies with 18 comparisons included in this study. Based on the TTC staining, G-Rd was found to have significant effects on diminishing the infarct size in the comparison with the control group which received normal saline or with no treatment. After excluding one study, which calculated the actual infarct volume only through deducting the area of brain edema [56], while others used the adjusted infarct volume and use the percentage of the contralateral structure to expression (19 studies, SMD = −1.75 [−2.21 to − 1.30], *P* < 0.00001) (Figure 3).

Subgroup analysis showed that the pooled estimates of infarct size improvement did not depend on the species, ischemic time, timing regimen, and so on, but was associated with the dose (Table 3). Subgroup analysis was conducted to identify G-Rd lower the cerebral infarct volume on experimental cerebral I/R. The results illustrated a dose-response relationship in a dose no more than 50 mg/kg, in the studies using doses of >10-<50 mg/kg (4 studies, SMD = −5.08 [−7.58 to − 2.58], *P* < 0.0001) is more preferable than less than 10 mg/kg (6 studies, SMD = −1.48 [−2.52 to − 0.45], *P* = 0.005), 10 mg/kg (6 studies, SMD = −2.25 [−3.00 to − 1.50], *P* < 0.00001), or 50 mg/kg (11 studies, SMD = −1.53 [−1.86 to − 1.21], *P* < 0.00001). But when the dose was greater than 50 mg/kg, the protection of G-Rd on reducing the cerebral infarct volume was ineffective (3 studies, SMD = −1.18 [−3.10 to 0.73], *P* = 0.22).

### 3.5. The Effective of G-Rd for Cerebral NFS in Cerebral I/R Damage and Meta-Analysis

**Figure 4:**
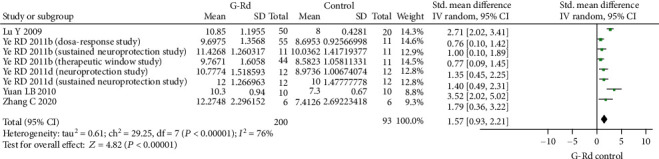
The pooled estimate of G-Rd in the improvement of neurological function score (higher score means better recovery). G-Rd: ginsenoside-Rd.

**Figure 5:**
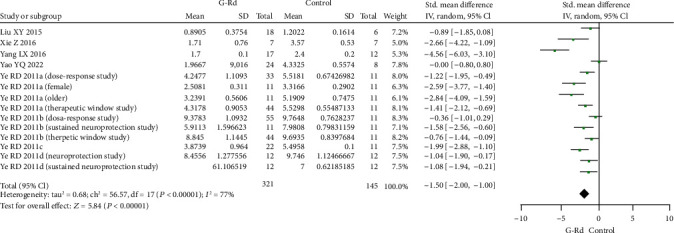
The pooled estimate of G-Rd in the improvement of neurological function score (lower score means better recovery). G-Rd: ginsenoside-Rd.

The reduction of infarct volume was associated with notable behavioral improvement. The NFS was still significantly improved with G-Rd treatment in the focal I/R injury setting. Twelve studies assessed neurological scores using different scoring systems. The 3-18 grading scale, which developed by Garcia et al. [61], was used in four studies [35, 46, 47, 58]. The 0-12 grading scale [35, 58], Zea-Longa score [22, 55, 57], modified neurological severity score [33, 45], Bederson's score [56], and other neurological scores [59] together to assess motor and sensory recovery after I/R injury. All of the included studies pointed the protective effect of G-Rd in improvement of the neurological deficits. In the scale category with a higher score indicating a better functional recovery (8 studies, SMD = 1.57 [0.93 to 2.21], *P* < 0.00001) (Figure 4). And in another scale category with a lower score indicating a better functional recovery into (14 studies, SMD = −1.50 [−2.00 to − 1.00], *P* < 0.00001) (Figure 5).

### 3.6. Publishing Bias Test

**Figure 6:**
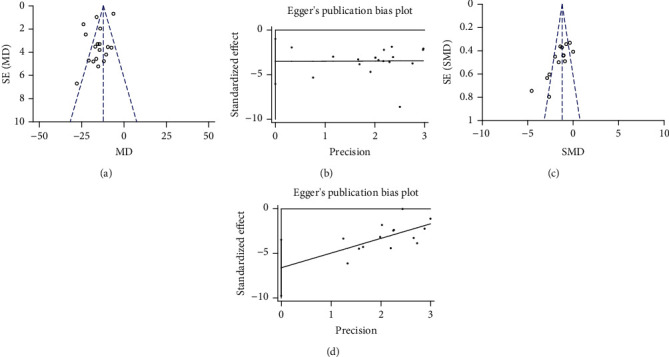
Bias assessment plot for the effect of G-Rd on infarct volume by funnel blot (a) and Egger's test (b); neurological function score (lower score means better recovery) by funnel blot (c) and Egger's test (d). G-Rd: ginsenoside-Rd.

The funnel plot test was used to check the meta-analysis publication bias, there was asymmetric for the effect of G-Rd on infarct volume (Figure 6(a)) and the funnel plot of the NFS (the lower score means better recovery) was essentially symmetrical (Figure 6(c)). Then, Egger's tests were conducted, the *P* values for the Egger's intercept suggested a moderate likelihood of publication bias for the effect of G-Rd on infarct volume analysis (*P* = 0.046 < 0.05) (Figure 6(b)), while the NFS (the lower score means better recovery) is with a low risk of publication bias for all analysis (*P* = 0.279 > 0.05) (Figure 6(d)).

## 4. Discussion

### 4.1. Summary of the Main Results

Systematic review and meta-analysis have already demonstrated the preclinical evidence that G-Rg1 and G-Rb1 have potential neuroprotective role in substantially reduced infarct volume and improved NFS in animal models of cerebral I/R injury [62, 63]. Up to now, this is the first meta-analysis to evaluate the promising therapeutic effect of G-Rd in focal brain I/R animal models. The qualities of the included studies were generally moderate. Evidence has showed that G-Rd treatment before and/or after I/R stroke can reduce infarct volume, enhance neurological function. Subgroup analysis showed that G-Rd in the range of >10-<50 mg/kg dose substantially lower the infarct size, while beyond this range the effect was abrogated. Maybe G-Rd at 50 mg/kg dose has reached the upper limit of the blood concentration, and too high a dose may produce drug toxicity. Among the G-Rd administration dose-response studies, two studies revealed that in their test paradigms treat with 40 mg/kg performed better [22, 47]. Lu Y et al. explained the protective effect decrease may be related to the increased dosages lead to the animals cannot tolerate the drug toxicity [47]. Some studies found that the reduction in infarct volume was greatest in 50 mg/kg, but their experimental was limited by a wide range of doses, they did not design a dose study between 10 and 50 mg/kg, and the result comes from the same teamwork [33, 45, 58]. Although >10-<50 mg/kg showed the best effect on reduce infarct volume, we also found that most of the studies involving dose-response relationship were not set the dose between >10-<50 mg/kg, which may affect the reliability of our conclusion. Refer to the subgroup analysis, further research about dose-response of G-Rd is needed at the concentrations ranging from 10 to 50 mg/kg to determine the optimal dose in the management of brain I/R damage.

### 4.2. Neuroprotective Strategies of G-Rd in Experimental I/R Injury Animal Model

G-Rd is the main bioactive saponins in Panax notoginseng and ginseng extracts. G-Rd contributes to neuroprotective with extensive biological activity. The possible mechanisms of G-Rd on focal cerebral ischemia animal models were discussed in this study (Table 4).

### 4.3. G-Rd Ameliorates ROS Production to Antioxidation on Cerebral I/R Damage to Impeded Injury

**Figure 7:**
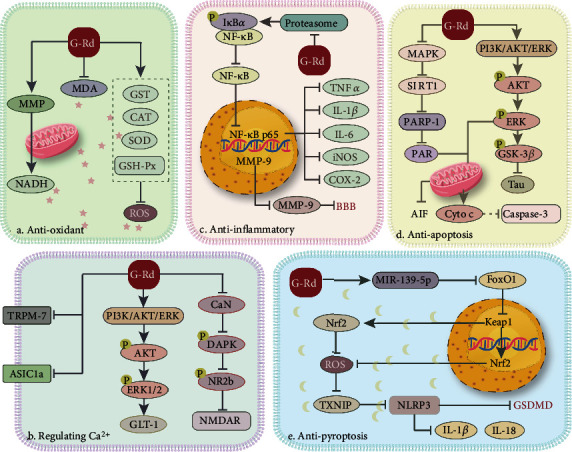
The neuroprotective mechanisms of G-Rd for I/R damage in experimental animal. MMP: mitochondrial membrane potential; NADH: nicotinamide adenine dinucleotide; MDA: malondialdehyde; GST: glutathione S-transferase; CAT: catalase; SOD: superoxide dismutase; GSH-Px: glutathione peroxidase; ROS: reactive oxygen species; I*κ*B*α*: nuclear factor of kappa light polypeptide gene enhancer in B cell inhibitor, alpha; NF-*κ*B: nuclear factor kappa-light-chain-enhancer of activated B cells; MMP-9: neuroinflammation-mediated matrix metalloproteases-9; TNF-*α*: tumor necrosis factor alpha; IL-1*β*: interleukin 1*β*; IL-6: interleukin 6; iNOS: inducible nitric oxide synthase; COX-2: cyclooxygenase-2; BBB: the blood-brain barrier; MAPK: mitogen-activated protein kinase; SIRT1: sirtuin1; PARP-1: poly (ADP-ribose) polymerase-1; PAR: poly(ADP-ribose); AIF: apoptosis-inducing factor; PI3K: phosphatidylinositol 3-kinase; Akt: proteinserine-threonine kinase; ERK: extracellular regulated protein kinases; GSK-3b: glycogen synthase kinase-3b; Cyto C: cytochrome c; GLT-1: glial glutamate transporter-1; CaN: calcineurin; DAPK: death-associated protein kinase; NR2b: N-methyl-D-aspartate receptor 2B; NMDAR: N-methyl-d-aspartate receptor; TRPM 7: transient receptor potential melastatin-7; ASIC1a: acid sensing ion channels 1a; FoxO1: forkhead box transcription factor O1; Keap1: Kelch-like ECH-associated protein 1; Nrf2: nuclear factor erythroid-2-related factor 2; TXNIP: thioredoxin-interacting protein; NLRP3: nucleotide-binding oligomerization domain- (NOD-) like receptor 3; GSDMD: gasdermin D; G-Rd: ginsenoside-Rd; I/R: ischemia/reperfusion.

Oxidative stress plays a crucial role in cerebral I/R damage [16]. ROS generation was start during ischemia and burst after reperfusion. Importantly, the chemical structure of G-Rd (with sugar moiety located at the 20th position of the triterpene dammarane) determines its direct antioxidant properties [64]. G-Rd directly impeded the inactivation of cerebral I/R-induced Nrf2/heme oxygenase-1(HO-1)/NAD(P)H: quinine oxidoreductase 1(NQO-1) antioxidant pathway inactivation [22]. The activation of the signaling pathway can induce protective genes through transcription to eliminate the production of reactive oxygen species, thereby resisting the oxidative stress injury caused by ischemia/reperfusion [65]. G-Rd inhibited the reduction of Nrf2, HO-1, and NQO-1, which further increased the superoxide dismutase (SOD) activity, and improve the level of glutathione peroxidase (GSH-Px) and catalase (CAT). In the ischemic penumbra, G-Rd dampened the accumulation of malondialdehyde (MDA) and 4-hydroxynonenal (4-HNE) to inhibit lipid peroxidation, G-Rd suppressed the expression of advanced glycosylation end products (AGEs) to alleviate protein denaturation, as well as it reduced 8-hydroxy-deoxyguanosine (8-OHdG^+^) to improve nucleic acid and DNA damage against neuron injury [33, 58]. Overwhelming evidence implied that G-Rd acted as an antioxidant also through protecting mitochondrial metabolism. G-Rd significantly attenuated the loss of aconitase to antioxidative stress damage [33, 35]. It might diminish the mitochondrial dysfunction significantly associated with mitochondrial membrane potential (MMP) hyperpolarization as well as the elevation of mitochondrial electron transport complexes activities to ameliorate ROS production [35, 58]. In addition, G-Rd lowers the accumulation of lactate and increase pyruvate, respectively, hence improving energy status (Figure 7(a)) [35].

### 4.4. G-Rd Regulating Ca^2+^ to Impeded Excitatory Toxicity after Cerebral I/R Damage

Many studies have shown that excitatory toxicity is the trigger of all downstream events. Glutamate-induced excitotoxicity is an important factor [66]. In the early stage of cerebral ischemic, glutamate is markedly elevated in the extracellular space [51]. Excessive glutamate leads the N-methyl-d-aspartate (NMDA) receptor overactivation, which results in cytosolic Ca^2+^ overload and triggers a cascade of molecular events. To modulate glutamate-/NMDA-induced Ca^2+^ influx, G-Rd significantly upregulates glial glutamate transporter glutamate transporter-1 (GLT-1) via the phosphatidylinositol 3-kinase (PI3K)/extracellular regulated protein kinases 1/2 (ERK1/2) pathways to promote glutamate clearance [51]. Meanwhile, G-Rd inhibited the phosphorylation of NMDA receptor 2B (NR2b) induced by cerebral ischemia to interfere the NMDA receptor function, whose overactivation-induced Ca^2+^ overload to causes nervous excitatory [48, 56, 59]. Zhang et al. investigated G-Rd attenuated death-associated protein kinase (DAPK1) by reducing calcineurin (CaN) activity-mediated NR2b phosphorylation [59]. G-Rd antagonizes the accumulated Ca^2+^ also via regulating the nonglutamate dependent calcium-permeable cation channels. Such as acid sensing ion channels (ASIC) and transient receptor potential (TRP). G-Rd enhanced ASIC2a, inhibited ASIC1a expression, and downregulated transient receptor potential melastatin-7 (TRPM7) to mediated neuroprotection (Figure 7(b)) [5, 49].

### 4.5. G-Rd Makes Effect on Anti-Inflammation to Impeded Injury after Cerebral I/R Damage

Sequential inflammatory response plays a critical role in the pathophysiology of acute cerebral ischemic. G-Rd has also been noted to mitigate the acute stage of cerebral ischemia inflammatory response. G-Rd decreased Iba-1-positive microglia contribute to neuron death via secreting the proinflammatory cytokines including interleukin 1*β* (IL-1*β*), interleukin 6 (IL-6), and tumor necrosis factor alpha (TNF-*α*) [33, 53], and lowered inducible nitric oxide synthase (iNOS) released from activated astrocyte to stop triggering a stronger inflammatory cascade [33]. The neuroprotection of G-Rd was related to the suppression of cyclooxygenase-2 (COX-2) enzyme concentrations targeted in the inhibition of arachidonic acid release and metabolism [33]. NF-*κ*b participates in these anti-inflammation progress, G-Rd pretreatment restored nuclear factor of kappa light polypeptide gene enhancer in B cell inhibitor alph (I*κ*B*α*) expression in the cytoplasm, reduced the phosphorylation of I*κ*B*α*, and blocked p65 nuclear translation by promoting the formation of I*κ*B*α*-p65 complex [52–54]. In addition, G-Rd makes effect on maintain the blood-brain barrier integrity, not only to prevent peripheral leukocyte infiltration but also reduce brain edema in aggravating neurological deficit, and the machine is considered to be mediated by suppressing the NF-*κ*b/neuroinflammation-mediated matrix metalloproteases-9 (MMP-9) pathway (Figure 7(c)) [52].

### 4.6. G-Rd Plays a Role in Antiapoptosis on Cerebral I/R Damage to Impeded Injury

Ischemia induced the elevation of intracellular ROS and Ca^2+^ levels leading the MMP to open, which enhanced the mitochondria permeability and many mitochondrial proapoptotic factors release are the important event leading to neuronal apoptosis [67]. The TUNEL-positive cell in the G-Rd treatment group was lowered significantly [35, 48, 57]. G-Rd counteracts apoptosis was related to the inhibition of apoptosis-inducing factor (AIF) and cytochrome c [35, 54]. The expression of cleaved caspase-3 significantly depressed in the G-Rd-treated rats compared with the control group [35, 57]. G-Rd management inhibits AIF release from mitochondria initiating the caspase-independent apoptotic cascade through the adenosine 5′-monophosphate-activated protein kinase/poly ADP-ribose polymerase-1 (AMPK/PARP-1) single pathway [35, 54]. G-Rd promoted the numbers of GFAP^+^ and DCX^+^ cells increased after focal I/R through the PI3K/Akt [55]. On the other hand, G-Rd serves as a promising drug to treat poststroke dementia by preventing the phosphorylation level of tau in the PI3K/AKT pathway (Figure 7(d)) [50].

### 4.7. G-Rd Anti-NLRP3-Mediated Pyroptosis on Cerebral I/R Damaged Tissues

Brain I/R damage involves a range of complex pathological mechanisms, and ROS trigger the NLRP3-mediated pyroptosis which has been implicated in cerebral I/R damage [22, 68]. The effective of antipyroptosis on G-Rd management has been investigated [22]. G-Rd promotes NLRP3 inflammasome inactivation by reducing ROS level to impeded procaspase 1 autocleaved into active caspase 1, which not only converts the precursors of IL-18 and IL-1*β* into their mature forms but also promotes the gasdermin D (GSDMD) maturation then causes the neuron pyroptosis [69]. G-Rd upregulated miR-139-5p to inhibit forkhead box transcription factor O1 (FOXO1), which regulates Kelch-like ECH-associated protein 1 (Keap1) transcriptional activity and subsequently triggers the Nrf2 antioxidant pathway. It is essential for the reduction of excessive ROS and inhibits the ROS/thioredoxin-interacting protein (TXNIP)/NLRP3 inflammasome axis-mediated pyroptotic in ischemic cortical tissues (Figure 7(e)).

Several limitations should be considered, and first of all, due to time constraints, the protocol of this study was not registered in any registration platform, which was important to restrict the likelihood of biased post hoc decisions in review methods. Secondly, despite the quality of the included studies was acceptable, there are still some shortcomings. For example, no studies included here have reported sample size calculation and allocation concealment. More than half of the studies did not report blind evaluation of results. Only a half study was clearly proposed how to define a completed stroke model according to neurological function score. Then ischemic stroke affects elder patients preferentially accompanied by multiple risk factors, such as diabetes, hypertension, hyperlipidemia, obesity [70, 71], and certain female-specific risk factors may explain their higher risk of stroke [72]. G-Rd was well tolerated and with no dose-related adverse event patterns were assessed in healthy volunteers [73]. Preliminary, multicenter randomized, double-blind, placebo-controlled, phase II clinical trials also have showed that G-Rd had significantly effective on reduced neurological deficits and improved the scores of National Institutes of Health Stroke Scale (NIHSS) [36, 37, 53]. Further investigations are needed to be included in clinical trials to validate the results.

## 5. Conclusion

Pooled data analysis from this study approved that treatment with G-Rd prior- and/or post-I/R reduced infarct volume and enhanced neurological functions in brain I/R injury animal model. The recommended dosage is >10- <50 mg/kg. Based on the literature data, G-Rd can reduce oxidative stress, antagonize the accumulated Ca^2+^, and inhibit inflammatory resistance to apoptosis and antipyroptosis on cerebral ischemia. In the future, the protective effects of G-Rd are needed to be confirmed in clinical trial.

## Figures and Tables

**Table 1:** Basic characteristics of the included studies.

Study	Animals	Model	No. of animals	Groups	Treatment	Assessment
Yao et al. [22]	Male C57BL/6 (22-25 g)	MCAO 1 h	8/8/8/8/8	1. Sham 2. MCAO+1,3-propanediol 3. MCAO+Rd (10 mg/kg) 4. MCAO+Rd (20 mg/kg) 5. MCAO+Rd (40 mg/kg)	0.5 h pre- and 2 post-MCAO, *i.p.*	IV, NF, edema, no. of neurons, caspase 1-TUNEL^+^, NLRP3, ASC, caspase 1, GSDMD-FL, GSDMD-N, IL-18, IL-1*β*, TXNIP, ROS, HO-1, NOQ1, Trx1, Keap1, FoxO1, nuclear Nrf2, TXNIP-NLRP3

Ye et al. [33]	Male SD (270-320 g)	MCAO 2 h	161 (total)	1. Sham 2. MCAO+VEC 3. MCAO+Rd (0.1 mg/kg) 4. MCAO+Rd (1 mg/kg) 5. MCAO+Rd (10 mg/kg) 6. MCAO+Rd (50 mg/kg) 7. MCAO+Rd (200 mg/kg)	0.5 h pre-MCAO, *i.p.*	IV, NF, 2,3-DHBA, 2,5-DHBA, 8-OHdG^+^, 4-HNE, MDA, AGEs, carbonyls, GPX, CAT, SOD1/2, GR, IBal^+^, GSH/GSSG, iNOS, COX-2

Ye et al. [35]	Male SD (270-320 g)	**A/B.** MCAO 2 h	**A/B.** 12/12 **C.** 6/6/6/6	**A/B. neuroprotection and sustained neuroprotection study**	**A**. 0.5 h pre-MCA **B**. 0.5 h pre-MCAO then continued until POD 7, *i.p.*	IV, NF, complex I~IV activity, MMP, ROS, aconitase activity, glucose, lactate, LPR, TUNEL^+^, AIF, pro-/cleaved caspase-3, pyruvate, Cyto C
				1. MCAO+VEC 2. MCAO+Rd (50 mg/kg)		

Ye et al. [45]	Male, female, and old male SD	**A/B/D.** MCAO 2 h **C.** MCAO 24 h	/	**A. Dose-response study**	**A.** 0.5 h pre-MCA **B/C.** 0, 2, 4, 8 h post- MCAO **D.** Post-MCAO, *i.p.*	IV, NF
				1. MCAO+VEC 2. MCAO+PBN 3. MCAO+Edaravone 4. MCAO+Rd (1 mg/kg) 5. MCAO+Rd (10 mg/kg) 6. MCAO+Rd (50 mg/kg)		
				**B/C. therapeutic window and permanent MCAO study**		
				1. MCAO+VEC 2. MCAO+Rd (50 mg/kg) (at different times)		
				**D. Female and aged rat study**		
				1. MCAO+VEC 2. MCAO+Rd (50 mg/kg)		

Yuan et al. [46]	Male SD (280-320 g)	MCAO 2 h	10/10/10/10/10/10	1. Sham 2. MCAO 3. MCAO+Rd (30 mg/kg) 4.MCAO+Rd+Pur 5.MCAO+Rd+TMP 6.MCAO+Rd+Pur+TMP	1 h pre-MCAO, *i.p.*	IV, NF

Lu et al. [47]	Male SD (280-320 g)	MCAO 2 h	10/10/10/10/10/10/10	1. MCAO 2. MCAO+VEC 3. MCAO+Rd (5 mg/kg) 4. MCAO+Rd (10 mg/kg) 5. MCAO+Rd (20 mg/kg) 6. MCAO+Rd (40 mg/kg) 7. MCAO+Rd (80 mg/kg)	1 h pre-MCAO, *i.p.*	IV, NF

Du et al. [48]	Male Wistar (250-300 g)	MCAO 1 h	9/27/27/27	1. Control 2. Sham 3. MCAO 4. MCAO+Rd (2 mg/kg)	3 d pre-MCAO, *i.p.*	IV, TUNEL, NR2B, EndoG

Zhang et al. [49]	Male SD (250-300 g)	MCAO 2 h	12/12/12/12	1. Sham 2. MCAO+VEC 3. Sham+Rd (10 mg/kg) 4. MCAO+Rd (10 mg/kg)	15-minute pre-MCAO, *i.p.*	TRPM7, ASIC1a, ASIC2a, NR1, NR2A, NR2B

Zhang et al. [50]	Male SD (270-320 g)	MCAO 2 h	15/15/15/6	1. Sham 2. MCAO 3. MCAO+Rd (30 mg/kg) 4. MCAO+LY294002+Rd (30 mg/kg)	1 h pre-MCAO and 10 mg/kg/d until sacrificed, *i.p.*	IV, NF, no. of neurons, S199/202, PHF-1, tau-5, p-GSK-3b, p-Akt

Zhang et al. [51]	Male SD (270-320 g)	MCAO 1.5 h	16/15/17	1. Sham 2. MCAO+SA 3. MCAO+Rd (30 mg/kg)	1 h pre-MCAO, *i.p.*	GR, GLT-1

Zhang [52]	Male SD (270-320 g)	MCAO 2 h	8/8/8/8	1. Sham 2. MCAO 3. MCAO+Rd (30 mg/kg) 4. MCAO+MG132	1 h pre-MCAO, *i.p.*	Cerebral edema, MMP-9, 20S proteasome activities, P65, NF-*κ*B, I*κ*B*α*, BBB function

Zhang et al. [53]	Male SD (250-300 g)	MCAO 2 h	/	1. Sham 2. MCAO+VEC 3. Sham+Rd (10 mg/kg) 4. MCAO+Rd (10 mg/kg)	4 h post-MCAO, *i.p.*	IBa1+, IL-1*β*, IL-6, IL-18, TNF-*α*, IFN-*γ*, p-I*κ*B*α*, I*κ*B*α*, nuclear and cytosolic p65

Hu et al. [54]	Male SD (280-300 g)	MCAO 2 h	10/30/10/30	1. Sham+SA 2. MCAO+SA 3. Sham+Rd (10 mg/kg) 4. MCAO+Rd (10 mg/kg)	0.5 h pre-MCAO, *i.p.*	PAR, PARP-1, nuclear p65, nuclear and mitochondria AIF

Liu et al. [55]	Male SD (220-240 g)	MCAO 1.5 h	19/19/9/9/19/10	1. Sham 2. MCAO 3. MCAO+Rd (1 mg/kg) 4. MCAO+Rd (2.5 mg/kg) 5. MCAO+Rd (5 mg/kg) 6. MCAO+LY294002+Rd (5 mg/kg)	POD1 to POD3, *i.p.*	IV, NF, p-Akt/Akt, GFAP, DCX^+^

Xie et al. [56]	Male SD (270-320 g)	MCAO 2 h	21/42/21/42	1. Sham+VEC 2. MCAO+VEC 3. Sham+Rd (50 mg/kg) 4. MCAO+Rd (50 mg/kg)	0.5 h pre-MCAO, *i.p.*	IV, NF, NR2B, p-Ser-1303, p-Tyr-1472, p-Ser-1480

Yang et al. [57]	Male SD (270-320 g)	MCAO 2 h	24/24/24/24	1. Sham 2. MCAO 3. MCAO+Rd (30 mg/kg)	1 h pre-MCAO and 10 mg/kg/d until sacrifice, *i.p.*	NF, NEIL1, NEIL2, NEIL3, cleaved caspase-3, TUNEL^+^, mtDNA, nDNA, survival rate

Ye et al. [58]	Male C57BL/6 (16-18-month)	MCAO 1 h	/	**A. Dose-response study:**	**A.** 0.5 h pre-MCA **B**. 0, 2, 4, 6, 8 h post-MCAO **C.** 0.5 h pre-MCAO then continued until POD 7, *i.p.*	IV, NF, body weight, MDA, 8-OHdG^+^, carbonyl levels, MMP, ROS, SOD1/2, CAT, GPX, GST, GSH/GSSG aconitase activity, complex I-IV activity
				1. MCAO+VEC 2. MCAO+Rd (0.1 mg/kg) 3. MCAO+Rd (1 mg/kg) 4. MCAO+Rd (10 mg/kg) 5. MCAO+Rd (50 mg/kg) 6. MCAO+Rd (200 mg/kg)		
				**B. Therapeutic window study**		
				1. MCAO+VEC 2. MCAO+Rd (50 mg/kg) (at different times)		
				**C. Sustained neuroprotection study**		
				1.MCAO+VEC 2. MCAO+Rd (50 mg/kg)		

Zhang et al. [59]	Male SD (280-300 g)	MCAO 2 h	20/20/20/20	1. Sham 2. MCAO 3. Sham+Rd (10 mg/kg) 4. MCAO+Rd (10 mg/kg)	Immediately post-MCAO, *i.p.*	IV, NF, p-ser-1303, p-tyr1472, p-ser1480, p-DAPK

MCAO: middle cerebral artery occlusion; Rd: ginsenoside Rd; i.p.: intraperitoneally; IV: infarct volume; NF: neurological functions; TUNEL: terminal deoxynucleotidyl transferase-mediated dUTP biotin nick end labeling; NLRP3: nucleotide-binding oligomerization domain- (NOD-) like receptor 3; ASC: apoptosis-associated speck-like protein containing a CARD; GSDMD: gasdermin D; IL-18: interleukin 18; IL-1*β*: interleukin 1*β*; TXNIP: thioredoxin-interacting protein; ROS: reactive oxygen species; HO-1: heme oxygenase-1; NOQ1: reduced coenzyme/quinone oxidoreductase 1; Trx1: thioredoxin; Keap1: Kelch-like ECH-associated protein 1; FoxO1: forkhead box transcription factor O1; Nrf2: nuclear factor erythroid-2-related factor 2; SD: Sprague-Dawley; VEC: vehicle; DHBA: dihydroxybenzoic acids; 8-OHdG: 8-hydroxy-deoxyguanosine; 4-HNE: 4-hydroxynonenal; MDA: malondialdehyde; AGEs: advanced glycosylation end products; GPX: glutathione peroxidase; CAT: catalase; SOD: superoxide dismutase; GR: glutathione reductase; GSH: glutathione; GSSG: glutathione disulfide; iNOS: inducible nitric oxide synthase; COX-2: cyclooxygenase-2; MMP: mitochondrial membrane potential; LPR: lactate/pyruvate ratio; AIF: apoptosis-inducing factor; Cyto C: cytochrome c; PBN: N-tert-butyl-alpha-phenylnitrone; Pur: puerarin; TMP: tetramethylpyrazine; NR2B: N-methyl-D-aspartate receptor 2B; Endo G: endonuclease G; TRPM 7: transient receptor potential melastatin-7; ASIC: acid sensing ion channels; NR1: N-methyl-D-aspartate receptor 1; NR2A: N-methyl-D-aspartate receptor 2A; PHF-1: pairedhelicalfilaments-1; GSK-3b: glycogen synthase kinase-3b; Akt: proteinserine-threonine kinase; SA: saline; GLT-1: glial glutamate transporter-1; MMP-9: neuroinflammation-mediated matrix metalloproteases-9; I*κ*B*α*: nuclear factor of kappa light polypeptide gene enhancer in B cell inhibitor: alpha; NF-*κ*B: nuclear factor kappa-light-chain-enhancer of activated B cells; BBB: the blood-brain barrier; IL-6: interleukin 6; TNF-*α*: tumor necrosis factor alpha; IFN-*γ*: interferon gamma; PAR: poly(ADP-ribose); PARP-1: poly (ADP-ribose) polymerase-1; GFAP: glial fibrillary acidic protein; NEIL: endonuclease VIII-like; GST: glutathione S-transferase; DAPK: death-associated protein kinase.

**Table 2:** Risk of bias of the included studies according to CAMARADES checklist.

Study	1	2	3	4	5	6	7	8	9	10	Score
Yao et al. [22]	√	√	√	/	√	√	/	/	√	√	7
Ye et al. [33]	√	√	/	/	√	√	/	/	/	/	4
Ye et al. [35]	√	√	/	/	√	√	/	/	/	/	4
Ye et al. [45]	√	√	√	/	√	√	√	/	/	√	7
Yuan et al. [46]	√	√	√	/	√	√	/	/	/	/	5
Lu et al. [47]	√	√	√	/	√	√	/	/	/	/	5
Du et al. [48]	√	√	√	/	/	√	/	/	/	/	4
Zhang et al. [49]	√	√	√	/	/	/	/	/	/	√	4
Zhang et al. [50]	√	√	/	/	/	√	/	/	/	√	4
Zhang et al. [51]	√	√	/	/	/	√	/	/	/	√	4
Zhang et al. [52]	√	/	/	/	/	√	/	/	/	√	3
Zhang et al. [53]	√	√	√	/	/	√	/	/	√	√	6
Hu et al. [54]	√	√	√	/	/	/	/	/	/	/	3
Liu et al. [55]	√	/	√	/	√	/	/	/	√	/	4
Xie et al. [56]	√	√	√	/	/	/	/	/	/	/	3
Yang et al. [57]	√	√	√	/	/	√	/	/	/	√	5
Ye et al. [58]	√	√	/	/	√	/	√	/	/	/	4
Zhang et al. [59]	√	√	/	/	/	√	/	/	/	/	3

(1) Publication in a peer-reviewed journal; (2) statement of control of temperature; (3) randomization to treatment or control; (4) allocation concealment; (5) blinded assessment of outcome; (6) no obvious intrinsic neuroprotective effect of anesthetic; (7) appropriate animal model such as aged, diabetic, or hypertensive; (8) sample size estimation; (9) compliance with animal welfare regulations; (10) declared any potential conflict of interest.

**Table 3:** Subgroup analysis of decrement in infarct volume with G-Rd.

Pooled estimates	No. of studies	Std. MD (95% CI)	*P* value	Subgroup (*P* value)
Species				<0.00001
SD rats	13	-1.84 [-2.41, -1.28]	<0.00001	
Wistar rats	1	-5.98 [-11.96, 0.01]	=0.05	
C57BL/6 mice	4	-1.44 [-2.12, -0.75]	<0.0001	
Dosage				<0.00001
<10 mg/kg	6	-1.48 [-2.52, -0.45]	=0.005	
10 mg/kg	6	-2.25 [-3.00, -1.50]	<0.00001	
>10-<50 mg/kg	4	-5.08 [-7.58, -2.58]	<0.0001	
50 mg/kg	11	-1.53 [-1.86, -1.21]	<0.00001	
>50 mg/kg	3	-1.18 [-3.10, 0.73]	=0.22	
Administration time				<0.00001
Before I/R	7	-2.28 [-3.43, -1.13]	=0.0001	
After I/R	7	-1.49 [-1.84, -1,14]	<0.00001	
Before and after I/R	4	-1.64 [-2.38, -0.91]	<0.0001	
Occlusion time				<0.00001
60 min	5	-1.51 [-2.24, -0.78]	<0.0001	
90 min	1	-2.27 [-3.43, -1.11]	=0.0001	
120 min	11	-1.77 [-2.35, -1.18]	<0.00001	
Permanent	1	-1.57 [-2.42, -0.71]	=0.0003	
Model animal				<0.00001
Normal male	13	-1.77 [-2.04, -1.50]	<0.00001	
Female	1	-1.02 [-1.92, -0.12]	=0.03	
Older male	4	-1.18 [-1.58, -0.79]	<0.00001	
Risk of bias				<0.00001
<5	7	-1.32 [-1.87, -0.77]	<0.00001	
≥5	4	-3.12 [-4.99, -1.24]	=0.001	

G-Rd: ginsenoside-Rd; SD: Sprague-Dawley; I/R: ischemia/reperfusion; MD: mean difference; CI: confidence interval.

**Table 4:** The neuroprotective mechanism of G-Rd *in vivo* in the treatment of cerebral I/R injury.

Study	Proposed mechanism	Outcome
Yao et al. [22]	Anti-inflammation and antioxidation	NLRP3, ASC, caspase 1, GSDMD-N, IL-18, IL-1*β*, TXNIP, ROS, Keap1, FoxO1, TXNIP-NLRP3↓; HO-1, NQO1, Trx1, nuclear Nrf2↑
Ye et al. [33]	Antioxidation and anti-inflammation	2,3-DHBA, 2,5-DHBA, 8-OHdG^+^, 4-HNE, MDA, AGEs, carbonyls, extracellular glutamate, SOD1, GPX, IBal^+^, iNOS, and COX-2↓; CAT, SOD2, GR, GSH/GSSG↑
Ye et al. [35]	Mitochondrial protection and antiapoptosis	Complex I-IV activity, ROS, lactate, LPR, cleaved caspase-3, Cyto C, AIF↓; MMP, aconitase activity, glucose, pyruvate↑
Du et al. [48]	Regulating Ca^2+^ and antiapoptosis	NR2B, EndoG↓
Zhang et al. [49]	Regulating Ca^2+^	TRPM7, ASIC1a↓; ASIC2a↑
Zhang et al. [50]	/	S199/202, PHF-1, and tau-5↓; p-GSK-3b and p-Akt↑
Zhang et al. [51]	Regulating Ca^2+^	Extracellular glutamate↓; GR, GLT-1↑
Zhang et al. [52]	Anti-inflammation	20S proteasome activities, nuclear P65, NF-*κ*bp65; MMP-9↓; I*κ*B*α* and BBB function↑
Zhang et al. [53]	Anti-inflammation	IL-1*β*, IL-6, IL-18, TNF-*α*, IFN-*γ*, p-I*κ*B*α*, and nuclear NF-*κ*b p65↓; I*κ*B*α* and cytosolic p65↑
Hu et al. [54]	Anti-inflammation and antiapoptosis	PAR, nuclear p65, nuclear AIF↓; AIF in mitochondria↑
Liu et al. [55]	Antiapoptosis	p-Akt/Akt, GFAP^+^, and DCX^+^↑
Xie et al. [56]	Antiapoptosis	NR2B, p-Ser-1303, p-Tyr-1472, and p-Ser-1480↓
Yang et al. [57]	Antiapoptosis and antioxidation	Cleaved caspase-3, mtDNA, and nDNA↓; NEIL1, NEIL2, and NEIL3↑
Ye et al. [58]	Antioxidation and mitochondrial protection	8-OHdG^+^, MDA, carbonyl levels, ROS, SOD1↓; MMP, SOD2, CAT, GPX, GST, and GSH/GSSG Aconitase activity, complex I-IV activity↑
Zhang et al. [59]	Regulating Ca^2+^	p-ser-1303, p-tyr1472, p-ser1480, p-DAPK↓

G-Rd: ginsenoside-Rd; I/R: ischemia/reperfusion; NR2B: N-methyl-D-aspartate receptor 2B; Endo G: endonuclease G; 8-OHdG: 8-hydroxy-deoxyguanosine; MDA: malondialdehyde; ROS: reactive oxygen species; SOD: superoxide dismutase; MMP: mitochondrial membrane potential; CAT: catalase; GPX: glutathione peroxidase; GST: glutathione S-transferase; GSH: glutathione; GSSG: glutathione disulfide; DHBA: dihydroxybenzoic acids; 4-HNE: 4-hydroxynonenal; AGEs: advanced glycosylation end products; iNOS: inducible nitric oxide synthase; COX-2: cyclooxygenase-2; GR: glutathione reductase; LPR: lactate/pyruvate ratio; Cyto C: cytochrome c; AIF: apoptosis-inducing factor; TRPM 7: transient receptor potential melastatin-7; ASIC: acid sensing ion channels; PAR: poly(ADP-ribose); GLT-1: glial glutamate transporter-1; PHF-1: pairedhelicalfilaments-1; GSK-3b: glycogen synthase kinase-3b; Akt: proteinserine-threonine kinase; GFAP: glial fibrillary acidic protein; NEIL: endonuclease VIII-like; IL-1*β*: interleukin 1*β*; IL-6: interleukin 6; IL-18: interleukin 18; TNF-*α*: tumor necrosis factor alpha; IFN-*γ*: interferon gamma; NF-*κ*b: nuclear factor kappa-light-chain-enhancer of activated B cells; I*κ*B*α*: nuclear factor of kappa light polypeptide gene enhancer in B cell inhibitor: alpha; DAPK: death-associated protein kinase; MMP-9: neuroinflammation-mediated matrix metalloproteases-9; BBB: the blood-brain barrier; NLRP3: nucleotide-binding oligomerization domain (NOD)-like receptor 3; ASC: apoptosis-associated speck-like protein containing a CARD; GSDMD: gasdermin D; TXNIP: thioredoxin-interacting protein; Keap1: Kelch-like ECH-associated protein 1; FoxO1: forkhead box transcription factor O1; HO-1: heme oxygenase-1; NQO1: NAD(P)H: quinine oxidoreductase 1; Trx1: thioredoxin; Nrf2: nuclear factor erythroid-2-related factor 2.

## Data Availability

All data included in this study are available upon request by contact with the corresponding author.

## Supplementary Materials

Supplementary Materials The search strategy of PubMed.
