# Supplementary material for: Neuroprotective Effect and Possible Mechanisms of Ginsenoside-Rd for Cerebral Ischemia/Reperfusion Damage in Experimental Animal: A Meta-Analysis and Systematic Review
Source: Oxid Med Cell Longev. 2022 Sep 1;2022:7650438. doi: 10.1155/2022/7650438 (PMC9458376; doi:10.1155/2022/7650438)
Supplement: Supplementary Materials — The search strategy of PubMed. [file 7650438.f1.docx]

**Supplementary material. The search strategy of Pubmed.**

(((((((((((((((((brain anoxia ischemia [Title/Abstract]) OR brain hypoxia ischemia [Title/Abstract]) OR brain ischaemia [Title/Abstract]) OR brain ischaemics [Title/Abstract]) OR brain ischemia [Title/Abstract]) OR brain ischemias [Title/Abstract]) OR cerebral anoxia ischemia [Title/Abstract]) OR cerebral ischaemia [Title/Abstract]) OR cerebral ischaemia hypoxia [Title/Abstract]) OR cerebral ischemia [Title/Abstract]) OR cerebral ischemias [Title/Abstract]) OR cerebral ischemia hypoxia [Title/Abstract]) OR ischemic stroke [Title/Abstract]) OR ischemized reperfusate [Title/Abstract]) OR ischemized reperfusion injury [Title/Abstract]) OR reperfusion injury [Title/Abstract]) OR stroke [Title/Abstract]) AND (((Ginsenoside [Title/Abstract]) OR Ginsenosides[Title/Abstract]) OR Ginsenoside Rd [Title/Abstract]).
